# Association Between Human Leukocyte Antigens and Graft-Versus-Host Disease Occurrence in Allogeneic Hematopoietic Stem Cell Transplantation – A 10-Year Experience on Iranian Patients

**DOI:** 10.34172/aim.2022.125

**Published:** 2022-12-01

**Authors:** Mohsen Hamidpour, Elham Roshandel, Haniyeh Ghaffari Nazari, Ghazaleh Sankanian, Hossein Bonakchi, Maryam Salimi, Sina Salari

**Affiliations:** ^1^Hematopoietic Stem Cell Research Center, Shahid Beheshti University of Medical Sciences, Tehran, Iran

**Keywords:** Allogeneic hematopoietic stem cell transplantation, Graft-versus-host disease, Human leukocyte antigen, Survival

## Abstract

**Background:**

Human leukocyte antigen (HLA) molecules mediate critical roles in determining responsiveness or non-responsiveness of the immune system, especially in transplantation. Some studies have shown a possible association between certain HLA alleles and some allogeneic hematopoietic stem cell transplantation (allo-HSCT) outcomes such as acute/chronic graft-versus-host disease (aGVHD/cGVHD) and overall survival (OS). In the current study, we investigated any possible association of HLA subclasses and acute/chronic GVHD occurrence as well as OS in patients receiving HLA-matched sibling allo-HSCT.

**Methods:**

We retrospectively evaluated the association of various HLA alleles with the incidence of aGVHD, cGVHD, and OS of 162 patients who received allo-HSCT from HLA-matched sibling between 2009-2018 at Taleghani hospital in Tehran.

**Results:**

We found that the incidence of aGVHD grades II-IV was higher among patients who had HLA-B*07 (*P*=0.031) and HLA-DRB1*07 (*P*=0.052). The presence of HLA-A*01 was associated with 4.5-fold greater odds of incidence in the extensive-type of cGVHD (*P*=0.009). Furthermore, HLA-A*03 (*P*=0.089), HLA-B*13(*P*=0.013), HLA-B*40 (*P*=0.042), HLA-DRB1*02 (*P*=0.074), and HLA-DRB1*04 (*P*=0.039) were associated with a lower rate of OS.

**Conclusion:**

This study suggests that certain HLA alleles might influence the incidence and severity of acute or chronic GVHD in the context of HLA-matched sibling allo-HSCT. In addition, some specific HLA alleles help predict OS in allo-HSCT recipients. These results might be helpful in estimating the incidence of aGVHD, cGVHD, and OS as well as designing personalized therapy.

## Introduction

 Allogeneic hematopoietic stem cell transplantation (allo-HSCT) is a promising therapy for many hematological malignancies and disorders.^1^ Despite the advances in pre- and post-transplant supportive care, allo-HSCT remains associated with substantial transplant-related mortality. Graft-versus-host disease (GVHD) represents the highest frequency in mortality following allo-HCT.^2^ Host tissue damage induced by conditioning regimens releases inflammatory cytokines and activates various subsets of immune cells, including antigen presenting cells, T cells, and natural killer cells, which are involved in the pathophysiology of GVHD.^3^ Donor immune cells that exist in grafts from different stem cell sources such as bone marrow, mobilized peripheral blood, and cord blood can induce GVHD reactions after transplantation. Moreover, allo-reactive T-cells are known as a dominant subset of immune cells to moderate GVHD. CD4 + T and CD8 + T cells are two main allo-reactive T cell subtypes which recognize exogenous and endogenous molecules presented on the major histocompatibility complex (MHC) class II and MHC class I molecules, respectively.^3^ MHC, also called human leukocyte antigen (HLA), is the most polymorphic protein in the body with many various subclasses in each population.^4^ Regarding their frequent expression in all nucleated cells and high immunogenicity, HLAs are the most important antigens in transplantation. In case of HLA miss-match transplantations, they can serve as a foreign antigen provoking immune reactions causing graft-failure or GVHD.^5^ Therefore, matched HLA antigens at A, B, C, DRB1, and DQB1 locus between recipient and donor as well as prescribing immunosuppressive regimens have been considered to limit GVHD occurrence.^6^ In spite of this strategy, GVHD occurs in approximately 30% of the patients who undergo allo-HSCT from HLA-matched related donors. This rate is roughly 70% for patients with HLA-matched unrelated donors.^7^ With regards to GVHD developing even in the HLA-identical sibling allo-HSCTs, some studies evaluated the possible association between the presence or absence of specific HLA antigens and GVHD incidence in HLA-matched related and unrelated donors. More frequent HLA alleles which either increase or decrease the incidence of GVHD in HLA-identical sibling transplantations were reported in various populations.^8,9^ However, some studies did not find any change in the risk of GVHD through HLA antigens after bone marrow transplantation from HLA-identical sibling.^10^ In the current study, we investigated any possible association of HLA subclasses and acute/chronic GVHD occurrence as well as OS in patients receiving HLA-matched sibling allo-HSCT.

## Materials and Methods

###  Study Design and Data Collection

 We revised the clinical data of patients who underwent allo-HSCT between 2009 and 2018 at Taleghani hospital, Tehran, Iran. Patients were excluded ifthey underwent a second HSCT or related-/unrelated-HLA-mismatched transplantation. One hundred and sixty-two patients were found who received allo-HSCT from HLA-matched siblings and were aged between 15–62 years. HLA alleles among GVHD and non-GVHD patients were assessed to find frequent alleles present in patients with GVHD. All relevant demographic and clinical data were obtained from clinical records.

###  HSCT Procedure and GVHD Diagnosis

 Transplant procedures, including peripheral blood hematopoietic stem cell collection, conditioning regimen, GVHD prophylaxis, and supportive care (anti-microbial prophylaxis and transfusion) were performed for all patients as previously published.^11-13^ All recipients received G-CSF-induced peripheral blood stem cells from full HLA-matched sibling donors.

 Conditioning regimens consisted of intravenous (IV) busulfan (0.8 mg/kg every 6 hours for 4 days) followed by either cyclophosphamide (60 mg/kg/d for 2 days) or fludarabine (30 mg/m^2^ of body surface area once a day for 5 days). The combination of fludarabine (30 mg/m^2^ for 5 days, IV), 1-(2-chloroethyl)-3-cyclohexyl-1-nitrosourea (CCNU) (100 mg/m^2^ for 2 days, oral) and melphalan (40 mg/m^2^ for one day, IV) was prescribed in patients with Hodgkin’s disease (HD) and non-Hodgkin’s lymphoma (NHL). In patients with aplastic anemia (AA) and Fanconi anemia (FA), the administrated conditioning regimen was cyclophosphamide (as mentioned above) and 1.5 mg/kg of anti-thymocyte globulin (ATG) for three days.

 GVHD prophylaxis containing cyclosporine A (CsA), and methotrexate (MTX) was given daily starting two days before transplantation. All patients received 3 mg/kg/d, IV of CsA from day -2 until + 5 (the day of allo-HSCT is considered day 0) followed by 12.5 mg/kg/d, P.O. until day + 180. Then, 10 mg/kg MTX, IV was added to the regimen from day + 1, with a dosage change to 6 mg/kg on days + 3, + 6 and + 11. In some patients, a dose of 1.5 mg/kg ATG, IV for 3 days (-3 until -1) was included in the combination.

 GVHD was diagnosed and graded according to the National Institutes of Health (NIH) and modified Glucksberg criteria^2,14^ and treated with adjusted CsA dose and methylprednisolone as the first line of treatment. Infection prophylaxis was comprised of acyclovir (antiviral), ciprofloxacin (antibacterial), and fluconazole (antifungal). Grade 0 and I of aGVHD was considered as no GVHD and mild GVHD with slight transient symptoms, respectively. Grade II-IV of aGVHD was considered as moderate to severe aGVHD. Extensive cGVHD was defined as generalized and/or localized skin involvement skin or liver dysfunction plus involvement of other target organs. The hemoglobin threshold was considered 7g/dL for RBC transfusion. The PLT cut-off for platelet transfusion was 20 × 10^9^/L.

###  HLA Typing

 The DNA-based HLA typing technique determined HLA allele types in both patients and donors. Genomic DNA was extracted from blood samples collected with EDTA by the sailing out method (Yekta Tajhiz, Iran).^15^ The quantification of DNAs was measured using a 260 nm spectrophotometer, and then DNA samples were stored at -70^o^C. Specific primer-polymerase chain reaction (SSP-PCR) (Olerup commercial kit, Sweden) was applied to characterize HLA-A, B, and DRB1in two digits. The SSP steps are as follows;

1- Initial denaturation for 2 minutes at 94°C
2- Denaturation for 10 seconds at 94°C
3- Annealing and extension for 1 minute at 65°C

Repeat steps 2 to 3, 10 times

4- Denaturation for 10 seconds at 94°C
5- Annealing for 50 seconds at 61°C
6- Extension for 30 seconds at 72°C

Repeat steps 4 to 6, 20 times

7- End-stage was held at RT if the process lasted less than 8 hours or at 4°C if longer than 8 hours. Final volume of PCR reactions was 10 µL

According to the manufacturer, agarose gel (2%) electrophoresis was used to resolve PCR products based on their molecular weight, visualized by staining with a safe stain under UV light, and photographed. SSP-PCR results were interpreted based on comparison with internal control relative sizes by data sheets of kit manufacture and software (SCORE version 6, Sweden).

###  Statistical Analyses

 Analysis was conducted to determine the effect of risk factors and HLA-alleles on the acute and chronic GVHD using a logistic regression model. Overall survival (OS) was defined as the time from HSCT to death for any reason. Moreover, an analysis was performed to identify the effects of HLA-alleles on time to event using the Cox proportional hazard model. Plotting the score process and Kolmogorov-type supremum test were applied to execute the proportional hazards assumption. The significance level was 0.05.

 In the univariable analysis for Cox and logistic regression, the significance level was set at 25%. In all of the multivariable analyses, a backward method with a significance level of 10% was utilized for selecting the highest predictive value features. The analysis was performed using the SAS program (version 9.4; SAS Institute Inc., Cary, NC, USA).

## Results

###  Patients and Donors

 The patients’ and donors’ clinical characteristics are shown in Table 1. The median age of the patients was 32.5 years (range: 1-62) of whom 83 (51.2%) and 78 (48.1%) were males and females, respectively. The median age of the donors was 32 years (range: 2-73) including 96 (59.35%) males and 64 (39.5%) females, respectively. Nine (5.6%), 10 (6.2%), 81 (50%), 42 (25.9%), 5 (3.1%) and 6 (3.7%) patients were diagnosed with NHL, HD, AML, ALL, AA and unclassified, respectively.

**Table 1:** Clinical and Demographic Characteristics of Patients (N = 162).

**Characteristics of Patients **	
Recipient age (y), Median (Rang), Missing	32.5 (15–62), 4 (2.5%)
Donor Age (y), Missing	32 (18–73), 46 (28.4%)
Male:Female, Missing	
Recipient	83 (51.2%):78 (48.1%), 1 (0.6%)
Donor	96 (59.3%):64 (39.5%), 2 (1.2%)
Gender match, Donor → Recipient	
Male → Male	45 (27.8)
Male → Female	49 (30.2)
Female → Female	26 (16)
Female → Male	39 (24.1)
Missing	3 (1.9)
Disease	
NHL	9 (5.6%)
HD	10 (6.2%)
AML	81 (50%)
ALL	42 (25.9%)
Aplastic/Fanconi anemia	5 (3.1%)
Unclassified	6 (3.7%)
Missing	9 (5.6%)
Recipient CMV IgG	
Positive	72 (44.4%)
Negative	11 (6.8%)
Missing	79 (48.8%)
Conditioning regimen	
Bu/Cy	85 (52.5%)
Bu/Flu	37 (22.8%)
Bu/Flu/ATG	15 (9.3%)
Flu/CCNU/Melphalan	19 (11.7%)
Cy/ATG	5 (3.1%)
Missing	1 (0.6%)
Prophylaxis regimen	
CSA + MTX	102 (63%)
CSA + MTX + ATG	16 (9.9%)
Missing	44 (27.2%)
Compatibility blood group	
Compatible	85 (52.5%)
Incompatible	71 (43.8%)
Missing	6 (3.7%)
Delivery history (n = 64*)	
Yes	23 (35.9%)
No	39 (60.9%)
Missing	2 (3.1%)
Acute GVHD	
0-I	123 (76%)
II-IV	30 (18.5%)
Missing	9 (5.5%)
Chronic GVHD	
No cGVHD	118 (72.8%)
Limited cGVHD	4 (2.5%)
Extensive cGVHD	5 (3.1%)
Missing	35 (21.6%)

*Number of Female Donors.

###  The Effect of HLA Alleles and Risk Factors on Acute and Chronic GVHD Incidence

**Figure 1:**
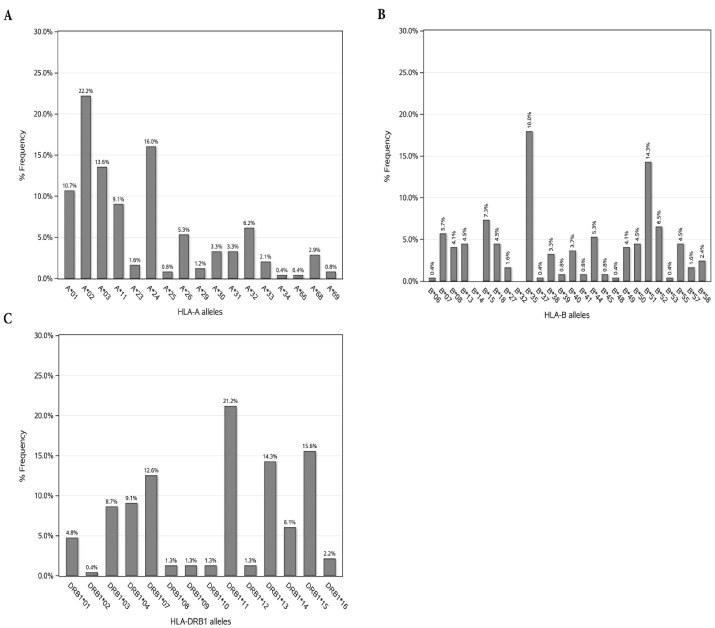
Frequency of HLA -A, -B, and -DRB1 Alleles in HSCT Patients with HLA-Identical Sibling Donors

 In this center, the incidence of acute and chronic GVHD were 30 out of 153 (19.6%) and 9 out of 127 (7.1%) available records. The frequency distribution of HLA variants in patients is illustrated in Figure 1. In HLA-A, the highest frequency pertained to A*02 (22.2%) and the lowest frequencies pertained to A*34 and A*66 with 0.4%. In HLA-B, the highest frequency pertained to B*35 (18%) and the lowest frequencies pertained to B*14, B*32 with 0% and B*06, B*37, B*48, B*53 with 0.4%.

 In HLA-DRB1, the highest frequency pertained to DRB1-11 (21.2%) and the lowest frequency pertained to DRB1-02 (0.4%). The results for the effects of HLA alleles and the risk factors on acute and chronic GVHD along with their cross tabulation with occurrence acute and chronic GVHD are shown in Tables 2 and 3, respectively. As stated in Table 2, based on the univariate model, 9 HLA alleles were significantly associated with the incidence of grades II-IV acute GVHD. Also, CMV serostatus, gender and gender disparity between donor and recipient were significantly related to the incidence of grades II-IV acute GVHD. HLA-A*29, HLA-B*07, HLA-B*18, HLA-B*57, HLA-DRB1*07 and HLA-DRB1*13 were associated with the increase in incidence of grades II-IV acute GVHD; among which, A*29 and B*57 alleles caused a greater increase in the odds of acute GVHD incidence compared to other alleles (OR: 2.76; 75% CI: 1.35-5.66; *P* = 0.098). HLA-B*51, HLA-B*52 and HLA-DRB1*15 had a significant decreasing impact on the incidence of grades II-IV acute GVHD. The CMV positive patients had 7% greater odds of acute GVHD incidence compared to CMV negative patients (OR: 1.07; 75% CI: 1.13-2.56; *P* = 0.131). The female patients, in comparison with males, had 30% greater odds of acute GVHD incidence (OR: 1.30; 75% CI: 1.03-1.65; *P* value = 0.194). Transplantations with a male donor and a female recipient had 71% greater odds of grades II-IV acute GVHD incidence compared to male-to-male allo-HSCTs [OR: 1.71; 75%CI:(1.18-2.48); *P* value = 0.089]. According to the multiple model, HLA-B*07 and HLA-DRB1*07 were significantly associated with the incidence of grades II-IV acute GVHD in which by holding the effect of other variables constant, the presence of HLA-B*07 was associated with 3.84-fold increased odds of incidence (AOR: 3.84; 90% CI: 1.37-10.72; *P* = 0.031). Additionally, HLA-DRB1*07 was associated with 2.11-fold increased odds of incidence (AOR: 2.11; 90% CI: 1.12-3.95; *P* = 0.052). As detailed in Table 3, based on the univariate model, seven HLA alleles were significantly associated with the incidence of the extensive-type of chronic GVHD. The presence of B*40, B*41, B*57 alleles increased the odds of extensive-type chronic GVHD incidence more than other alleles (OR: 4.69; 75% CI: 1.97-11.13; *P* = 0.029]. Based on the multiple model, HLA-A*01 was significantly associated with the incidence of chronic GVHD and the patients with A*01 allele had 4.5-fold greater odds of extensive-type incidence of chronic GVHD [OR: 4.50; 90% CI: (1.74-11.64); *P* = 0.009].

**Table 2:** Association of HLA Alleles and Risk Factors with Acute Graft-Versus-Host Disease

**Variables**	**aGVHD Grades 0-I (n=123)**	**aGVHD Grades II-IV (n=30)**	**Univariate**		**Multiple**^a^	
			** OR (75% CI)**	***P*** ** value** ^*^	**AOR (90% CI)**	***P*** ** value** ^**^
HLA-A29						
A29 +	1 (33.3)	2 (66.7)	2.76 (1.35-5.66)	0.098		
A29- (RL)	88 (79.3)	23 (20.7)	1			
HLA-B07						
B07 +	7 (53.8)	6 (46.2)	1.92 (1.35-2.73)	0.031	3.84 (1.37-10.72)	0.031
B07- (RL)	82 (81.2)	19 (18.8)	1		1	
HLA-B18						
B18 +	7 (63.6)	4 (36.4)	1.49 (1.01-2.19)	0.231		
B18 - (RL)	82 (79.6)	21 (20.4)	1			
HLA-B51						
B51 +	25 (89.3)	3 (10.7)	0.59 (0.40-0.86)	0.108		
B51- (RL)	64 (74.4)	22 (25.6)	1			
HLA-B52						
B52 +	14 (93.3)	1 (6.7)	0.47 (0.25-0.86)	0.152		
B52- (RL)	75 (75.8)	24 (24.2)	1			
HLA-B57						
B57 +	1 (33.3)	2 (66.7)	2.76 (1.35-5.66)	0.103		
B57- (RL)	88 (79.3)	23 (20.7)	1			
HLA-DRB1*07						
DRB1*07 +	17 (68)	8 (32)	1.38 (1.03-1.85)	0.198	2.11 (1.12-3.95)	0.052
DRB1*07- (RL)	69 (80.2)	17 (19.8)	1		1	
HLA-DRB1*13						
DRB1*13 +	18 (66.7)	9 (33.3)	1.45 (1.09-1.93)	0.121		
DRB1*13- (RL)	68 (81)	16 (19)	1			
HLA-DRB1*15						
DRB1*15 +	28 (84.8)	5 (15.2)	0.72 (0.52-0.98)	0.228		
DRB1*15- (RL)	58 (74.4)	20 (25.6)	1			
Recipient CMV IgG						
Positive	56 (83.6)	11 (16.4)	1.07 (1.13-2.56)	0.131		
Negative (RL)	7 (63.6)	4 (36.4)	1			
Recipient gender						
Female	57 (76)	18 (24)	1.30 (1.03-1.65)	0.194		
Male (RL)	65 (84.4)	12 (15.6)	1			
Gender match						
Female → Female	22 (84.6)	4 (15.4)	0.79 (0.47-1.32)	0.242		
Female → Male	28 (77.8)	8 (22.2)	1.24 (0.82-1.88)	0.589		
Male → Female	33 (71.7)	13 (28.3)	1.71 (1.18-2.48)	0.543		
Male → Male	37 (30.8.4)	5 (16.7)	1	0.089		

RL, reference level; AOR, adjusted odds ratio; aGVHD, acute graft-versus-host disease. ^a^ Backward Selection; *Significant at 0.25; **Significant at 0.10.

**Table 3:** Association of HLA Alleles and Risk Factors with Chronic Graft-Versus-Host Disease

**Variables**	**cGVHD 0-Limited (n=122)**	**cGVHD Extensive (n=5)**	**Univariate**		**Multiple**^a^	
			** OR (75% CI)**	***P*** ** value** ^*^	**OR (90% CI)**	***P*** ** value** ^**^
HLA-A01						
A01 +	15 (78.9)	4 (21.1)	4.44 (2.28-8.62)	< 0.001	4.50 (1.74-11.64)	0.009
A01- (RL)	74 (98.7)	1 (1.3)	1		1	
HLA-B07						
B07 +	5 (83.3)	1 (16.7)	2.04 (1.02-4.10)	0.228		
B07- (RL)	84 (95.5)	4 (4.5)	1			
HLA-B40						
B40 +	1 (50)	1 (50)	4.69 (1.97-11.13)	0.029		
B40- (RL)	88 (95.7)	4 (4.3)	1			
HLA-B41						
B41 +	1 (50)	1 (50)	4.69 (1.97-11.13)	0.029		
B41- (RL)	88 (95.7)	4 (4.3)	1			
HLA-B57						
B57 +	1 (50)	1 (50)	4.69 (1.97-11.13)	0.029		
B57- (RL)	88 (95.7)	4 (4.3)	1			
HLA-DRB1*08						
DRB1*08 +	2 (66.7)	1 (33.3)	3.24 (1.51-6.95)	0.072		
DRB1*08- (RL)	84 (95.5)	4 (4.5)	1			
HLA-DRB1*16						
DRB1*16 +	4 (80)	1 (20)	2.26 (1.11-4.59)	0.178		
DRB1*16- (RL)	82 (95.3)	4 (4.7)	1			

RL, reference level; OR, Odds ratio; cGVHD, chronic graft-versus-host disease. ^a^ Backward Selection; *Significant at 0.25; **Significant at 0.10.

###  The Effect of HLA Alleles on the Overall Survival

**Figure 2:**
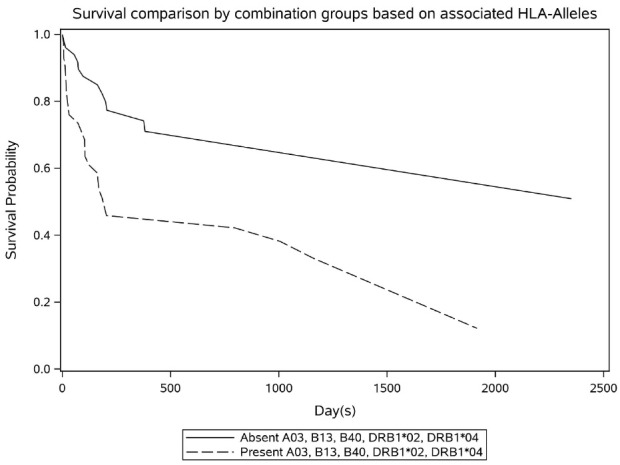
Survival Trend Based on Important HLA-Allele Variants

 The influence of various HLA alleles on the OS are shown in Table 4. According to the Cox univariate model, 10 HLA alleles were significantly associated with mortality rate. The presence of A*66 in HLA-A was responsible for the greatest increase in the mortality rate (HR: 3.88; 75% CI: 1.19–12.63; *P* value = 0.182). Moreover, in HLA-B, B*40 and B*41 were accountable for the highest increase in the risk of death (HR: 4.71; 75% CI: 2.01–11.03; *P* value = 0.028). The presence of DRB1*02 in HLA-DRB1 caused the greatest increase in mortality rate (HR: 7.67; 75% CI: 2.30–25.50; *P* = 0.049). The patients with B*38 allele had a 72% decrease in the risk of mortality (HR: 0.28; 75% CI: 0.08–0.92; *P* = 0.221). The proportional hazards assumption based on the score process plot and the Kolmogorov-type supremum test was established for HLA-alleles in the final multivariable model and this model was valid. Regarding the final multivariable model, HLA-A*03, HLA-B*13, HLA-B*40, HLA-DRB1*02, and HLA-DRB1*04 were significantly linked to death rate and increased mortality risk in patients (Figure 2).

**Table 4:** Association of HLA Alleles with Overall Survival

**Variables**	**Univariate**		**Multiple**^a^	
	**HR(75% CI)**	***P*** ** value** ^*^	**AHR(90% CI)**	***P*** ** value** ^**^
HLA-A03				
A03 +	1.99(1.36–2.93)	0.029	1.88(1.01–3.52)	0.089
A03- (RL)	1		1	
HLA-A31				
A31 +	2.58(1.25–5.29)	0.118	-	-
A31- (RL)	1		-	
HLA-A66				
A66 +	3.88(1.19–12.63)	0.183	-	-
A66- (RL)	1		-	
HLA-B07				
B07 +	2.01 (1.27–3.19)	0.068	-	-
B07- (RL)	1		-	
HLA-B13				
B13 +	2.16 (1.29–3.61)	0.081	3.06 (1.39–6.72)	0.013
B13- (RL)	1		1	
HLA-B38				
B38 +	0.28 (0.08–0.92)	0.221	-	-
B38- (RL)	1		-	
HLA-B40				
B40 +	4.71 (2.01–11.03)	0.028	4.85 (1.35–17.36)	0.042
B40- (RL^1^)	1		1	
HLA-B41				
B41 +	4.71 (2.01–11.03)	0.028	-	-
B41- (RL)	1		-	
HLA-DRB1*02				
DRB1*02 +	7.67 (2.30–25.50)	0.049	7.12 (1.19–42.62)	0.074
DRB1*02- (RL)	1		1	
HLA-DRB1*04				
DRB1*04 +	2.17 (1.41–3.32)	0.034	2.15 (1.13–4.10)	0.039
DRB1*04- (RL)	1		1	

RL, reference level; AHR, adjusted hazard ratio. ^a^ Backward Selection; *Significant at 0.25; **Significant at 0.10.

## Discussion

 Donor-recipient HLA disparity, age and gender of both the recipient and the donor, multiparous female donors as well as graft source, GVHD prophylaxis regimens, and intensity of conditioning regimen are known as risk factors of GVHD occurrence.^16^ In this study, we focused on the HLA alleles frequency in the Iranian allo-HSCT patients and examined the association between the presence or absence of specific HLA alleles and incidence of acute and/or chronic GVHD in allo-HSCT patients with an HLA-matched sibling donor. We determined that acute GVHD grade II-IV occurrence was remarkably increased in the presence of HLA-B*07 and HLA-DRB1*07. In addition, it was observed that HLA-A*01 was related to a greater risk of chronic GVHD incidence. Furthermore, we analyzed the correlation of HLA alleles with OS. As exemplified in the results, the presence of any alleles of HLA-A*03, -B*13, B*40, -DRB1*02, or -DRB1*04 predicted a decline in the OS rate. Shawkatová and colleagues reported that patients who carry HLA-A*01, -DRB1*03 and -DQB1*03 alleles had lower incidence of aGVHD. In contrast, the HLA-DQB1*06 allele increased the incidence of cGVHD.^17^

 Identification of the specific HLA alleles, associated with increasing or decreasing incidence ofacute or chronic GVHD and OS may have specific clinical implications in each population. Hence, the association of HLA loci or haplotypes with allo-HSCT outcomes have been evaluated in different populations. Storbet al estimated that the presence of HLA-B*18 is associated with a nearly three-fold increase in aGVHD incidence, whereas in patients with HLA-B*8 or HLA-Bw35, the incidence of aGVHD decreased by half, in comparison with those without these alleles.^18^ The European group for Blood and Marrow Transplantation (EBMT) conducted a cohort study in CML patients who received transplantation from HLA-matched sibling donors. In this homogenous large study, HLA-A*3 and HLA-DR1 significantly raised and diminished the occurrence of aGVHD grade II-IV, respectively.^19^ As our results suggested, HLA-B*7 was the HLA allele with the highest risk of aGVHD grade II-IV. Remberger et al^20^ pointed out that the HLA alleles A*10 and B*7 had a significant effect on enhancing the risk of acute GVHD grades II-IV. The chronic GVHD incidence was also low in patients who had the HLA-B27 allele. Ghavamzadeh et al reported an increased risk of severe acute grade III and IV GVHD in patients with HLA-DRB1*07. Our result confirmed this association in a way that these alleles could increase the risk of aGVHD grades II-IV by more than two times. Moreover, they suggested a protective role against severe a-GVHD for HLA-B35 in 162 recipients of HLA-identical sibling donor with different diseases.^21^ Given that the population ethnicity and sample size was similar to our study, some parallel/contradictory findings need further confirmation. Separate studies by the same center confirmed that the presence of HLA-A*11 and HLA-A*26 increased the risk of aGVHD, whereas HLA-A*3 reduced its risk in thalassemic patients who underwent HLA-Identical HSCT.^22^ A single-center experience in Korea demonstrated that the incidence of grades II to IV aGVHD was higher in patients with HLA-B*61 and HLA-Cw3. HLA-B*54 had close relationship with a higher incidence of extensive-type cGVHD. Furthermore, evaluating the association between HLA alleles and organ-related GVHD showed HLA-B*35, HLA-B*54, and HLA-B*7, B*27 to be associated with the development of severe acute skin GVHD, chronic skin/oral GVHD, and chronic liver GVHD, respectively. They suggested that these remarkable HLA alleles may be potent transplantation immune regulators.^8^ In a Brazilian population, increased incidence of acute GVHD grade III or higher was positively correlated with HLA-B*35, B*49, B*55 and in patients with extensive chronic GVHD, HLA-A*9, A*24 and A*26 were higher than other patients, while HLA-A*2 was lower.^9^

 Racial difference is one of the main factors that lead to the discrepancy between various HLA alleles linked to the risk of GVHD in different studies. In the context of HLA-matched transplantation, the role of specific HLA alleles in decreasing or increasing the incidence of GVHD could be explained by different mechanisms. One possible mechanism is the nature of the HLA system function in determining immune responses. As we know, the specificity of HLA–peptide–TCR interactions is fundamental in the function of the adaptive immune system.^23^ HLA molecules play a substantial role in mounting an efficient and appropriate response to non-self antigens and maintaining self-tolerance to prevent autoimmune diseases.^24^ Therefore, the presence or absence of various HLA molecules might determine responsiveness and/or unresponsiveness of the immune system against foreign antigens. For instance, HLADRB1*15 is known as a marker of clinical response to immunosuppressive therapy in autoimmune cytopenia.^25^ Battiwalla *et al.* reported that the presence of HLA-DRB1*15 in patients with myeloid malignancies was associated with a significantly lower incidence of aGVHD. Accordingly, we found a decreased incidence of aGVHD in HLA-DRB1*15 positive patients. There is a hypothesis that HLA-DRB1*15 could be a marker for a generalized susceptibility to immunosuppression.^26^

 Another possible mechanism for explicating GVHD occurrence in patients who receive HLA-matched transplantation is donor-recipient minor histocompatibility antigens (MiHAs) disparity. MiHAs are polymorphic peptide fragments which are derived from intracellular proteins. Despite the fact that MiHAs inheritance is independent of HLA molecules, they are presented by MHC class I and II and can be recognized by alloreactive T cells leading to induction of immune responses.^27^ It has been opined that various HLA alleles may have different capabilities to present MiHA that are responsible for GVHD in HLA-matched transplantation. A study reported some minor H and HY antigens which can induce T-cell responses more often than the others.^28^ Some of these H antigens (HA-1 and HA-2) are HLA-A*0201-restricted antigens which are solely expressed in hematopoietic-originated cells. Mismatching of HA-1 between donor-recipient in the context of HLA-matched bone marrow transplant was significantly associated with acute GVHD.^29^ Recently, MiHAs-based immunotherapy strategies have been considered as an interesting candidate not only to prevent GVHD development but also for inducing an effective GVT response.^30^

 On the other hand, some inflammatory mediators including histamine, prostaglandin, corticosteroids and cytokines, especially tumor necrosis factor alpha (TNF-α) which are pivotal in the pathophysiology of GVHD are encoded by HLA-related genes.^31^ Bouma et al demonstrated that the HLA-DR1-related TNF haplotype presents a low-secretor phenotype and produces lower levels of TNF-α in comparison with other HLA alleles. Consequently, reducing TNF-α secretion might provoke the HLA-DR1 protective effect on GVHD occurrence.^32^

 In conclusion, our data suggests that HLA alleles have independent influential effects on the occurrence of GVHD and also in OS of patients undergoing allo-HSCT, even in patients receiving the graft from an HLA-matched sibling donor. This might allude that certain HLA-antigens could act as an immunogenic stimulus to prompt the occurrence of GVHD and probably change the survival rate of patients. Furthermore, it is possible that MiHAs which are restricted to specific HLA alleles contribute to the incidence and severity of GVHD and HSCT outcome. Finding the contributing HLA alleles to the increased or decreased risk of GVHD and mortality might assist in selecting the strategies for prevention/treatment of GVHD and improve survival rate in HLA-matched HSCT patients. Our study was limited by the relatively small number of patients, absence of HLA-C, DR, and DQ typing for all patients who underwent HLA-matched sibling allo-HSCT between 2009 and 2018 at Taleghani hospital, and a the single-center nature of the study. Confirmation of the findings needs larger multi-center studies encompassing more HLA alleles and even MiHAs.
